# Coexpression of cellulases in *Pichia pastoris* as a self-processing protein fusion

**DOI:** 10.1186/s13568-015-0170-z

**Published:** 2015-12-23

**Authors:** Juliana de Amorim Araújo, Túlio César Ferreira, Marciano Régis Rubini, Ana Gilhema Gomez Duran, Janice Lisboa De Marco, Lidia Maria Pepe de Moraes, Fernando Araripe Gonçalves Torres

**Affiliations:** Departamento de Biologia Celular, Instituto de Ciências Biológicas, Universidade de Brasília, Brasília, DF 70910-900 Brazil

**Keywords:** Endoglucanase, Cellobiohydrolase, Protein fusion, 2A peptide, *Pichia pastoris*

## Abstract

The term cellulase refers to any component of the enzymatic complex produced by some fungi, bacteria and protozoans which act serially or synergistically to catalyze the cleavage of cellulosic materials. Cellulases have been widely used in many industrial applications ranging from food industry to the production of second generation ethanol. In an effort to develop new strategies to minimize the costs of enzyme production we describe the development of a *Pichia pastoris* strain able to coproduce two different cellulases. For that purpose the *eglII* (endoglucanase II) and *cbhII* (cellobiohydrolase II) genes from *Trichoderma reesei* were fused in-frame separated by the self-processing 2A peptide sequence from the foot-and-mouth disease virus. The protein fusion construct was placed under the control of the strong inducible *AOX1* promoter. Analysis of culture supernatants from methanol-induced yeast transformants showed that the protein fusion was effectively processed. Enzymatic assay showed that the processed enzymes were fully functional with the same catalytic properties of the individual enzymes produced separately. Furthermore, when combined both enzymes acted synergistically on filter paper to produce cellobiose as the main end-product. Based on these results we propose that *P. pastoris* should be considered as an alternative platform for the production of cellulases at competitive costs.

## Introduction

Cellulases represent a complex group of enzymes that act synergistically on cellulosic material to release sugars (Lynd et al. 2002). These biocatalysts account for a significant fraction of the market of industrial enzymes being extensively used in many industrial activities such as cotton processing, paper recycling, detergent formulation, juice extraction, among others (Bhat and Bhat 1997; Kumar et al. 2008, Wilson 2009). Cellulolytic enzymes are hydrolases that cleave *O*-glycosidic bonds and are classified according to the sites in which they act on the cellulosic substrate: endoglucanases, cleave internal bonds of the cellulosic fiber; exoglucanases, act in the reducing or non-reducing ends; and β-glucosidase, hydrolyze soluble oligosaccharides into glucose (Lynd et al. 2002). Filamentous fungi, especially those belonging to the genus *Aspergillu*s, *Trichoderma*, *Humicola*, *Penicillium* and *Phanerochaete*, are the microorganisms generally used as a source of cellulases (Singhania et al. 2010). Cellulases have also been produced in bacteria (Wood and Ingram 1992; Zhou and Ingram 2001) and yeast (Lynd et al. 2002, Cho and Yoo 1999, Fujita et al. 2002, 2004) as an alternative way of reducing production costs.

The methylotrophic yeast *Pichia pastoris* has become a host system widely used for the expression of a great number of heterologous proteins. The keys of its success have been widely reported in the literature and the advantages of using this yeast as expression platform include: GRAS (Generally Recognized as Safe) status, easy molecular genetic manipulation, high level production of secreted proteins, ability to promote post-translational modifications of higher eukaryotic and a preference for a respiratory rather than a fermentative metabolism to grow (Macauley-Patrick et al. 2005; Cregg et al. 2000, 2002; Hohenblum et al. 2004; Ahmad et al. 2014). These characteristics allow the production of large amounts of heterologous protein with relative technical facility and at costs lower than those of most other eukaryotic systems such as mammalian cell culture (Gellissen 2000, Higgins and Cregg 1998). Moreover, *P. pastoris* is capable of growing to very high cell densities using minimal media (Wegner 1990) and integrative vectors help to maintain the genetic stability of the recombinant elements even in large scale fermentation processes (Romanos et al. 1992). The promoter of alcohol oxidase 1 gene (*AOX1*) is the most commonly used in commercial expression vectors for directing expression of heterologous genes in *P. pastoris* since it is efficient and highly regulated by methanol (Yu et al. 2013; Lünsdorf et al. 2011; Sigoillot et al. 2012).

As in *Saccharomyces cerevisiae*, several microbial cellulases genes have been cloned and expressed in *P. pastoris* generally as individual expression cassettes (Valencia et al. 2014; Ramani et al. 2015; Salinas et al. 2011). One strategy to optimize the production of more than one protein in the same host is the construction of protein fusions which may be separated by a linker bearing the recognition site for a protease in order to promote the proteolytic cleavage of protein partners (Torres et al. 2010; Osborn et al. 2005; De Felipe et al. 2006). The use of the 2A sequence derived from FMDV (foot-and-mouth disease virus) is an alternative strategy that has been used to create multicystronic constructs capable of generating different proteins derived from a fusion protein precursor (Osborn et al. 2005). Initial studies showed that a sequence comprised of a region of 18 amino acid residues from 2A followed by a proline from protein 2B are enough to promote cleavage in a cotranslational manner. When this oligopeptide sequence is inserted between reporter genes the artificial polyprotein is efficiently cleaved in a manner analogous to FMDV (Ryan et al. 1991; Ryan and Drew 1994). Also, it was shown that the addition of 14 amino acid residues or more of the capsid protein 1D to the N-terminal of 2A increases the activity of self-cleavage up to 99 % (Donnelly et al. 2001a). After processing, the 2A peptide remains as a C-terminal extension of the upstream protein and all products downstream of 2A contain a proline residue at the N-terminal (Ryan et al. 1991; Donnelly et al. 2001b; De Felipe et al. 2003). So far, in all tested eukaryotic cells, including *P. pastoris*, it was shown that the cleavage mediated by the 2A sequence occurred with success (Lee et al. 2012; Rasala et al. 2012; Chng et al. 2015, Yen and Scheerlinck 2013; Luke et al. 2010; Wang et al. 2007; Roongsawang et al. 2010; Sun et al. 2012).

Aiming at the reduction of costs for cellulase production in this work we sought to use *P. pastoris* as a host for the production of an endoglucanase (EGII/Cel5A) and a cellobiohydrolase (CBHII/Cel6A) from *Trichoderma reesei* as a protein fusion separated by the 2A peptide. The catalytic properties of the processed protein partners were investigated in order to assess the use of this strategy to produce cellulases in *P. pastoris*.

## Materials and methods

### Strains and culture conditions

*Escherichia coli* DH5α was used for cloning and plasmid manipulation. This strain was grown in LB medium (0.5 % yeast extract, 1 % peptone and 1 % NaCl) supplied with 100 μg/ml ampicillin at 37 **°**C. *Pichia pastoris* GS115 (Invitrogen) was used as host for cellulase production. This strain was routinely grown in YPD medium (1 % yeast extract, 2 % peptone and 2 % glucose) at 30 **°**C. *Trichoderma reesei* RUT C-30 (ATCC 56765) was the source of cellulase genes and was cultivated at 30 **°**C in cellulase induction medium (1 % CMC—carboxymethyl cellulose, 1 % Sigmacel, 7.5 % salt solution [0.004 % Na_2_B_4_O_7_·10H_2_O, 1 % MgSO4·7H_2_O, 7.6 % KH_2_PO_4_] and 5 % trace elements [0.0026 % KCl, 0.04 % CuSO_4_·5H_2_O, 0.0714 % FeSO_4_·7H_2_O, 0.08 % Na_2_MoO_4_·2H_2_O, 0.0008 % ZnSO_4_·7H_2_O]).

### DNA procedures

All molecular cloning techniques were carried out as described elsewhere (Sambrook and Russell 2001). Restriction enzymes were obtained from New England Biolabs and used as detailed by the manufacturer. Primers were supplied by Integrated DNA Technologies (IDT).

### RNA isolation

For RNA isolation, *T. reesei* RUT C-30 was cultured on cellulase induction medium for 48 h at 30 **°**C and 220 rpm. The fungal mycelium was collected by filtration on sterile filter paper and 2 to 5 g (wet weight) was used for RNA extraction using the RNAeasy Mini Kit (Qiagen) following the manufacturer’s recommendations.

### Gene amplification

**Fig. 1:**
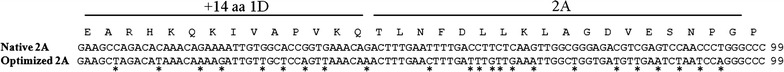
Alignment and predicted translation of the native and optimized nucleotide sequences of the 1D-2A region of FMDV. Base positions with an *asterisk* correspond to modifications that were made for codon optimization

Cellulase cDNA was obtained by RT-PCR from total RNA, using Superscript III First-Strand Synthesis System for RT-PCR kit (Invitrogen) as specified by the supplier. The DNA fragment encoding the *T. reesei* RUT C-30 *cbhII* gene (GenBank accession# M16190.1) was amplified from cDNA by PCR using primers cbhF (5′-GAGATCTAAAATGATTGTCGGCATTCTCACCACG) and cbhR (5′-GACTAGTCAGGAACGATGGGTTTGCGTTTG) which contain restriction sites (underlined) for *Bgl*II and *Spe*I, respectively. The amplicon represents the sequences that encode mature CBHII with its native signal peptide but lacking the translation stop codon. The *eglII* gene (GenBank accession # DQ178347) was amplified using primers 2A-egl2F (5′-GTCTAGA**GAAGCTAGACATAAACAAAAGATTGTTGCTCCAGTTAAACAA***ACTTTGAACTTTGATTTGTTGAAATTGGCTGGTGATGTTGAATCTAATCCAGGGCCC*ATGAACAAGTCCGTGGCTCCATTG) and egl2R (5′-GGCGGCCGCTTACTTTCTTGCGAGACACGAGCT) which contain *Xba*I and *Not*I restriction sites (underlined), respectively. Also, the 5′ end of primer 2A-egl2F contains the 2A sequence (italics) proceeded by 42 nucleotides (bold) representing the viral DNA downstream context (C-terminal region from protein 1D) (access number ACC63461.1). The amplicon represents the sequences that encode mature EGII with its native signal peptide including the translation stop codon. The codons for the 2A and 1D regions were adapted to the codon usage of *S. cerevisiae* to optimize expression in yeasts (Fig. 1). PCR was performed with Phusion DNA polymerase (Thermo Scientific) under the following cycling parameters: 30 cycles of 98 °C for 10 s, 59 °C for 1 min and 72 °C for 1 min, with a first denaturation step at 98 °C for 30 s and a final extension step at 72 °C for 10 min. Reaction mixtures contained 1X Phusion HF buffer, 200 mM each dNTPs, 0.5 μM each primers, 10 ng template and 0.02 U Phusion DNA polymerase in a final volume of 50 μL.

### Plasmid construction

**Fig. 2:**
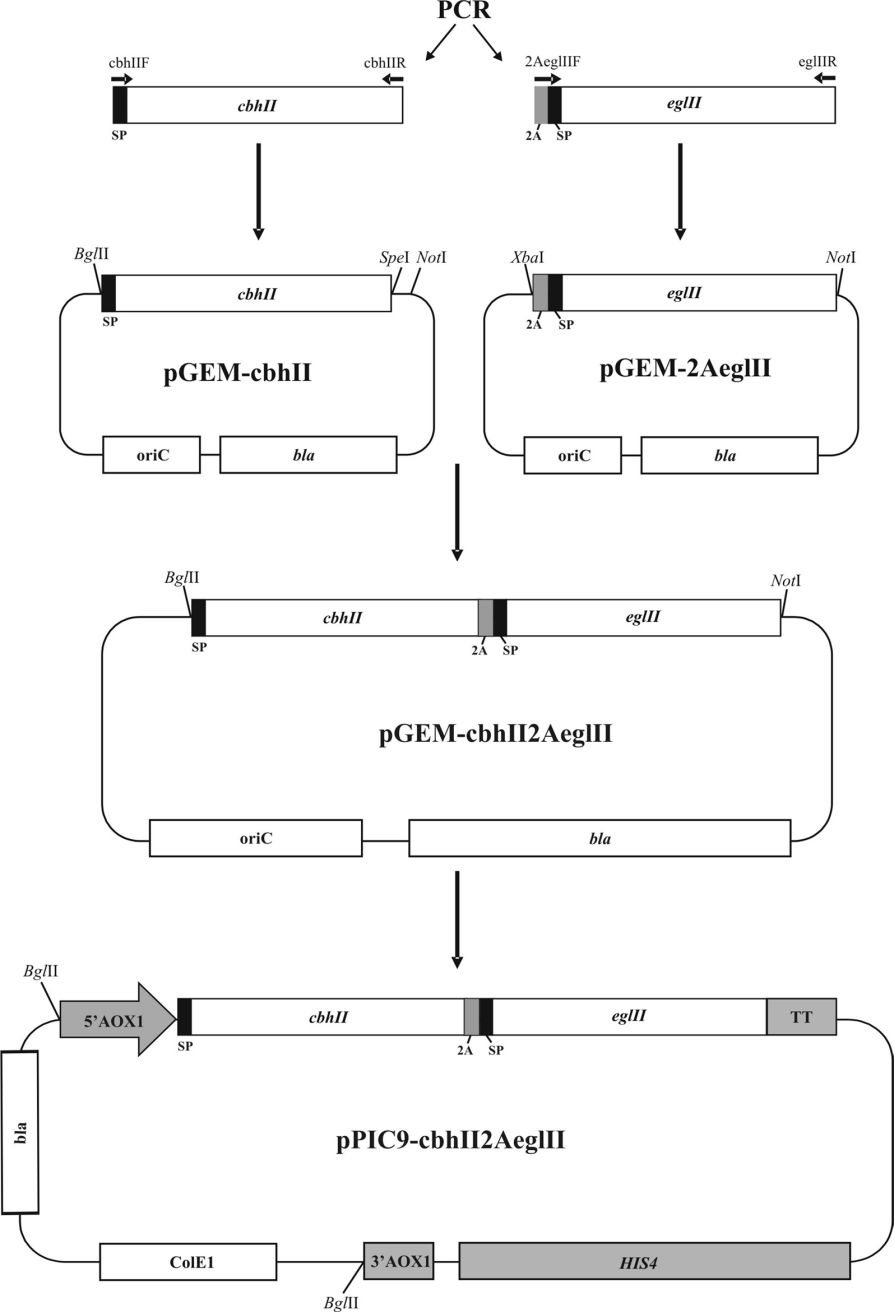
Schematic diagram showing the strategy for construction of the expression vector pPIC9-cbhII2AeglII. Vector construction is described in “Materials and methods”. *SP* native signal peptide, *2A* self-processing linker sequence, *5′AOX* 5′ promoter region of *AOX1*, *3′AOX1* 3′ sequences of *AOX1*, *TT* transcription termination sequence of *AOX1*

The strategy used to construct the vector for expression of the fusion protein described in this work is depicted in Fig. 2. The amplified cellulase cDNA products were cloned into pGEM-T vector to generate plasmids pGEM-2AeglII and pGEM-cbhII. Plasmid pGEM-2AeglII was digested with *Xba*I and *Not*I, and the 2A::*eglII* fragment was cloned into pGEM-cbhII digested with *Spe*I and *Not*I. The resulting vector was named pGEM-cbhII2AeglII. To obtain the yeast expression vector, pGEM-cbhII2AeglII was digested with *Bgl*II and *Not*I and the cassette containing the gene fusion was cloned into the *P. pastoris* expression vector pPIC9 digested with *Bam*HI and *Not*I which removes the original MFα signal peptide sequences. The protein fusion cassette was cloned under the control of inducible *AOX1* promoter and the resulting expression vector was named pPIC9- cbhII2AeglII.

Individual cellulase cDNA’s were also cloned into pPIC9: *cbhII* was amplified with primers cbhF and cbhRS (5´-GGCGGCCGCTTACAGGAACGATGGGTTTGCGT, *Not*I site is underlined) and *eglII* with primers egl2F (5´-GGGATCCAAAATGAACAAGTCCGTGGCTCCATTG, *Bam*HI site is underlined) and egl2R. Both amplicons were cloned as *Bgl*II-*Not*I or *Bam*HI-*No*tI inserts into pPIC9 digested with *Bam*HI and *Not*I resulting in plasmids pPIC9-cbhII and pPIC9-eglII.

### Transformation of *P. pastoris*

All recombinant plasmids derived from pPIC9 were used to transform *P. pastoris* GS115 by electroporation after linearization with *Bgl*II. Electrocompetent cells were prepared from a culture growing in log phase, mixed with 10 µg of linearized DNA and electroporated under the conditions described in the *Pichia* Expression Kit Instruction Manual (Invitrogen). Transformants were selected for their ability to grow on MD plates (1.34 % yeast nitrogen base, 4 × 10^−5^ % biotin, 2 % dextrose) lacking histidine at 30 °C. Vector pPIC9 was also used to transform *P. pastoris* to be used as negative control.

### Screening for cellulase activity

Individual transformed colonies were picked from MD plates and transferred to minimal methanol plates (100 mM potassium phosphate buffer [pH 6.0], 1.34 % yeast nitrogen base, 4 × 10^−5^ % biotin, 0.5 % methanol) containing 0.5 % CMC. Cells were grown at 30 °C for 72 h with the addition of 125 μL 100 % methanol on the internal part of the plate lid every 24 h. Plates were stained with 0.2 % Congo red solution for 15 min and dye excess was removed with 1 M NaCl. Cellulase-producing clones were identified by the presence of a hydrolysis halo around the colony (pPIC9-eglII and pPIC9- cbhII2AeglII). Because cells producing CBHII produce a halo that cannot be distinguished from that produced by the negative control the supernatant from clones transformed with pPIC9- cbhII were grown under inductive conditions and analyzed by SDS-PAGE. Clones producing EGII, CBHII, and the protein fusion were named EGLAOX, CBHAOX and FUSAOX, respectively.

### Protein production

Clones EGLAOX, CBHAOX and FUSAOX were grown in 5 mL BMGY medium (1 % yeast extract, 2 % peptone, 100 mM potassium phosphate buffer [pH 6.0], 1.34 % yeast nitrogen base, 4 × 10^−5^ % biotin, 1 % glycerol) in a 50 mL conic tube with constant shaking (200 rpm) at 30 °C for 24 h (OD_600_ ~ 12–15). The culture was collected by centrifugation at 1500×*g* for 5 min at room temperature and the cell pellet was resuspended with 10 mL BMMY medium (1 % yeast extract, 2 % peptone, 100 mM potassium phosphate buffer [pH 6.0], 1.34 % yeast nitrogen base, 4 × 10^−5^ % biotin, 0.5 % methanol) and grown under the same conditions. Methanol was added to the culture every 24 h to a final concentration of 0.5 % (v/v) to induce protein expression. At 24 h intervals after initiating induction, 1.0 mL aliquots of the culture were collected and the supernatants were immediately frozen and stored for further analysis.

### Protein purification

A 100 mL-culture of clone FUSAOX was grown under inductive conditions as described previously. The supernatant was concentrated and desalted against Milli Q water on an Amicon ultrafiltration membrane (10 kDa cut-off) at 4 °C. The pH was adjusted with 100 mM Tris–HCl (pH 7.5) and then loaded onto a prepacked anion exchanger column (Q-Sepharose, Hitrap Q-FF, 1 mL) equilibrated with five volumes of 50 mM Tris–HCl (pH 7.5) which was assembled on the Äkta Pure 25 System (GE Healthcare). Elution was performed on a linear gradient of NaCl (0–1 M) (20 column volumes) at a flow rate of 1 mL/min. Fractions of 0.5 mL were collected, dialyzed and tested for cellulolytic activity. Selected samples were analyzed on 12 % SDS-PAGE.

### Electrophoretic analysis

One milliliter of cell-free culture extract was precipitated by adding 250 μL 100 % TCA. After incubation for 3 h at 4 °C, the solution was centrifuged at 8000×*g* for 30 min. The pellet was washed with 1 mL ice-cold acetone and centrifuged under the same conditions. The pellet was dried at room temperature and dissolved in 20 μL 2X Laemmli sample buffer. The sample was boiled for 5 min and loaded on a 12 % SDS-PAGE. Proteins were visualized after staining with Coomassie brilliant blue R-250.

### Endoglycosidase digestion

One milliliter culture supernatant was precipitated and resuspended in 30 μL water. In order to analyze the presence of carbohydrates we added 2 µL peptide *N*-glycosidase F (PNGase F, Sigma) which removes *N*-glycosylations. The digestion was performed according to manufacturer’s recommendations. Deglycosylated proteins were analyzed by SDS-PAGE.

### Enzyme assay

Enzymatic activity was determined using filter paper as substrate and hydrolysis products were analyzed by high-performance liquid chromatography (HPLC). Assays were performed in reactions mixtures containing 500 μL culture supernatant, 1 mL 0.05 M sodium citrate buffer (pH 4.8) and filter paper (1 × 6 cm). The mixture was incubated at 50 °C for ~16 h and then analyzed on HPLC. All assays were performed in triplicate. The reaction mixtures were filtered through a 0.22 µm Millex PVDF membrane (Merck) and applied on an Aminex HPX-87p 300 × 7.8 mm column (Bio-Rad). Elution was performed at 60 °C with water as mobile phase at a flow rate of 0.6 mL/min and detection was monitored by refractive index RID (Shimadzu). A cellodextrin mixture consisting of glucose, cellobiose, cellotriose and cellotetraose (Sigma) was used as standard. Cellobiose production was calculated based on a standard curve with cellobiose concentrations ranging from 0.125 to 5.0 g/L. Protein concentration was determined by using the Bio-Rad Protein Assay, based on the Bradford dye-binding procedure using bovine serum albumin as standard.

## Results

**Fig. 3:**
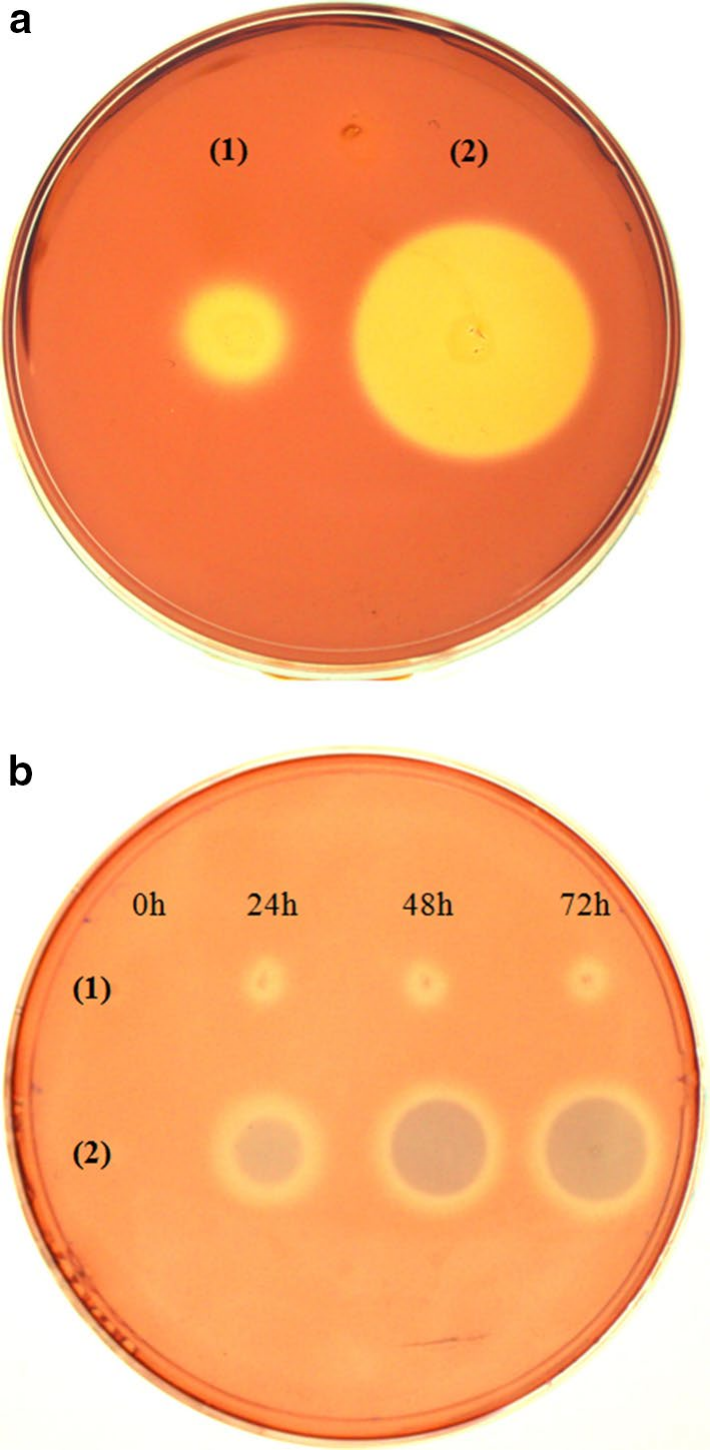
Plate assay for detection of cellulase activity in transformed *P. pastoris* cells. CMC agar plates were stained with Congo Red and enzyme activity is detected by the presence of a halo around the colony. **a** *P. pastoris* colonies grown for 72 h under methanol induction. **b** Supernatants (2 μL) from induced cultures collected at different times spotted directly onto CMC plate. *(1)* Recombinant yeast transformed with pPIC9 (negative control); *(2)* clone FUSAOX

**Fig. 4:**
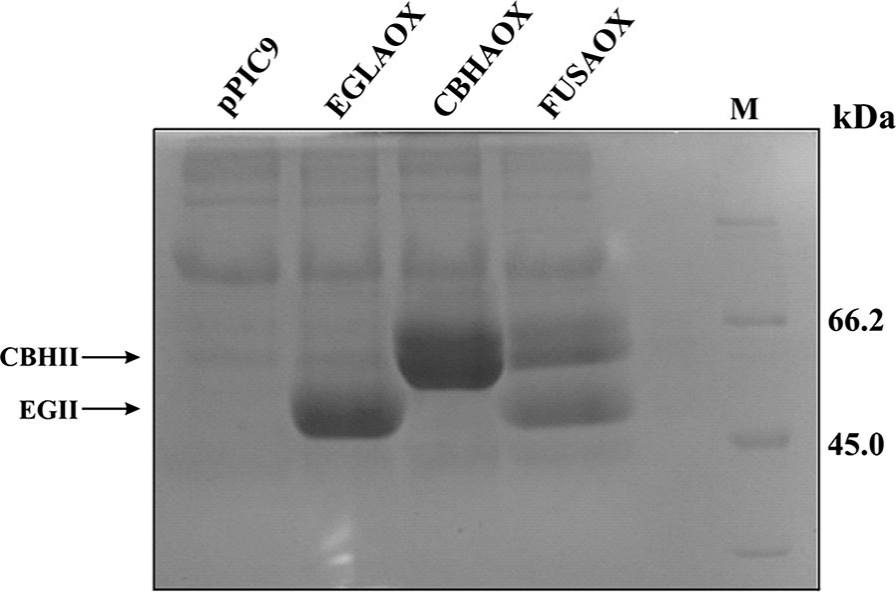
SDS-PAGE analysis of cell-free supernatants of recombinant yeasts induced with 0.5 % methanol for 72 h. (1) pPIC9 (negative control); (2) clone EGLAOX; (3) clone CBHAOX; (4) clone FUSAOX; M: Unstained protein molecular weight marker (Thermo Scientific). The position of the bands corresponding to CBHII and EGII is indicated by *arrows*

**Fig. 5:**
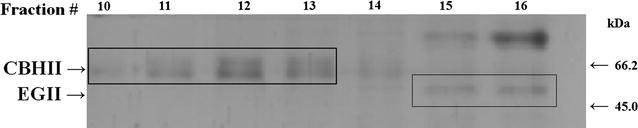
Purification of individual enzymes after protein fusion cleavage. The supernatant from clone FUSAOX was applied on a Q-Sepharose and proteins were eluted on a NaCl gradient. Sample fractions were analyzed on 12 % SDS-PAGE. CBHII was eluted on fractions 10–13 and EGII on fractions 15 and 16. The *rectangles* indicate individual enzymes. Fractions 12 and 16 were tested for cellulase activity. Only relevant bands of the unstained protein molecular weight marker (Thermo Scientific) are indicated by *arrows* at the *right side* of the panel

A gene fusion consisting of *cbhII* and *eglII* genes from *T. reesei* separated by the 2A sequence was cloned into the *P. pastoris* expression vector pPIC9 (Fig. 2). The resulting plasmid, pPIC9-cbhII2AeglII, was linearized with *Bgl*II to promote plasmid integration into the *AOX1 locus*. *P. pastoris* transformants were selected by the ability to grow on minimal agar medium lacking histidine due to the presence of the histidinol dehydrogenase gene (*HIS4*) in the transforming vector. The integration of the recombinant expression cassette into the yeast genome was confirmed by PCR using the same primers used for gene amplification (data not shown). Transformants were randomly selected and inoculated onto a plate containing CMC to detect secreted cellulase activity based on the formation of a hydrolysis halo. One clone named FUSAOX (Fig. 3a) was selected and to detect enzyme production it was grown in liquid medium under inductive conditions. The supernatants from the time course experiment were spotted onto a CMC-agar plate and the formation of halos with increasing sizes was observed (Fig. 3b). This confirmed that the enzymes produced by FUSAOX were secreted to the medium and not associated to the cell-wall. Furthermore, in order to detect the recombinant enzymes, culture supernatant collected after a 3-day induction with methanol were analyzed by SDS-PAGE. As controls, we used the supernatants from a yeast strain transformed with pPIC9 (Fig. 4, lane 1) and strains producing EGII (clone EGLAOX) and CBHII (clone CBHAOX) individually (Fig. 4, lanes 2 and 3, respectively). The supernatant from clone FUSAOX showed two major induction bands of equal intensities and with apparent molecular masses similar to those of the individual enzymes produced by clones EGLAOX (~50 kDa) and CBHAOX (~60 kDa) (Fig. 4, lane 4), thus confirming that the protein fusion had been successfully processed with no detected unprocessed products (~120 kDa). To further show that the proteins corresponded to active cellulases the supernatant from clone FUSAOX was fractionated by ion-exchange chromatography and the individual protein species were separated (Fig. 5) and shown to exhibit cellulase activity on CMC (data not shown).

**Fig. 6:**
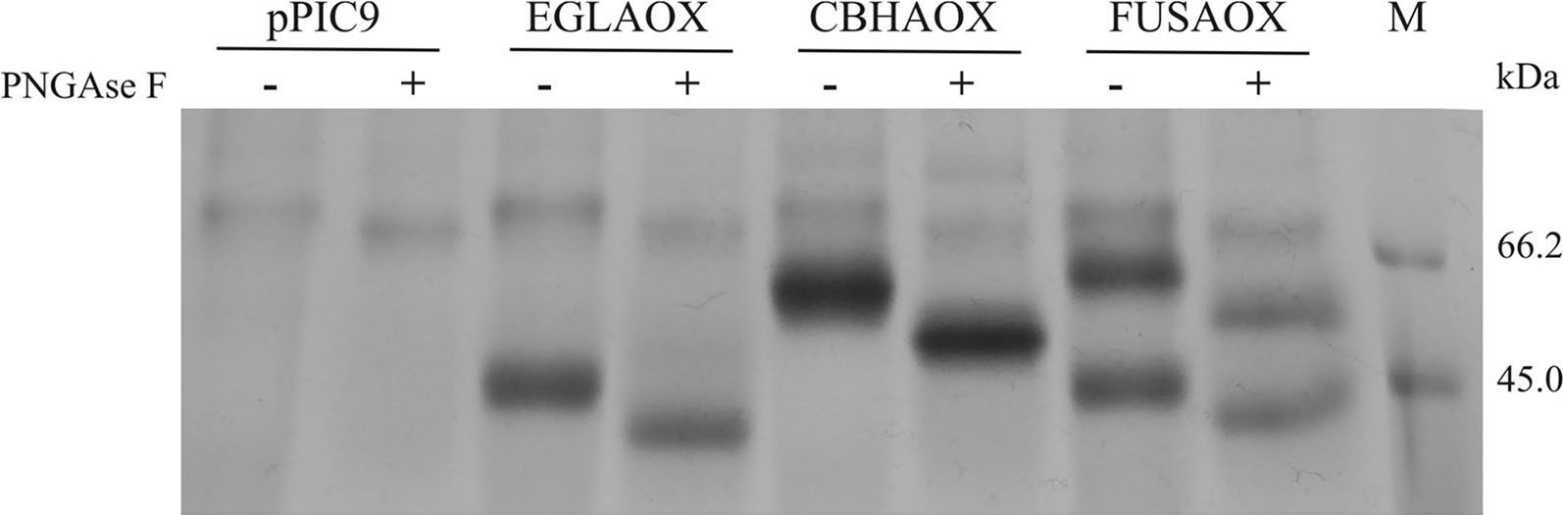
Deglycosylation test. Culture supernatants from the negative control (pPIC9) and clones EGLAOX, CBHAOX and FUSAOX were treated (+) or not (−) with PNGase F and analyzed on 12 % SDS-PAGE. M: Unstained protein molecular weight marker (Thermo Scientific)

To verify if the two cellulases produced in *Pichia* were glycosylated we performed a deglycosylation test with PNGase F which specifically removes *N*-linked sugars. When treated in such a way, the individual enzymes produced by clones EGLAOX and CBHAOX showed a reduction in the apparent molecular mass (Fig. 6, lanes 3–6) thus confirming protein glycosylation. A similar pattern was observed for the enzymes produced by clone FUSAOX (Fig. 6, lanes 7 and 8).

**Fig. 7:**
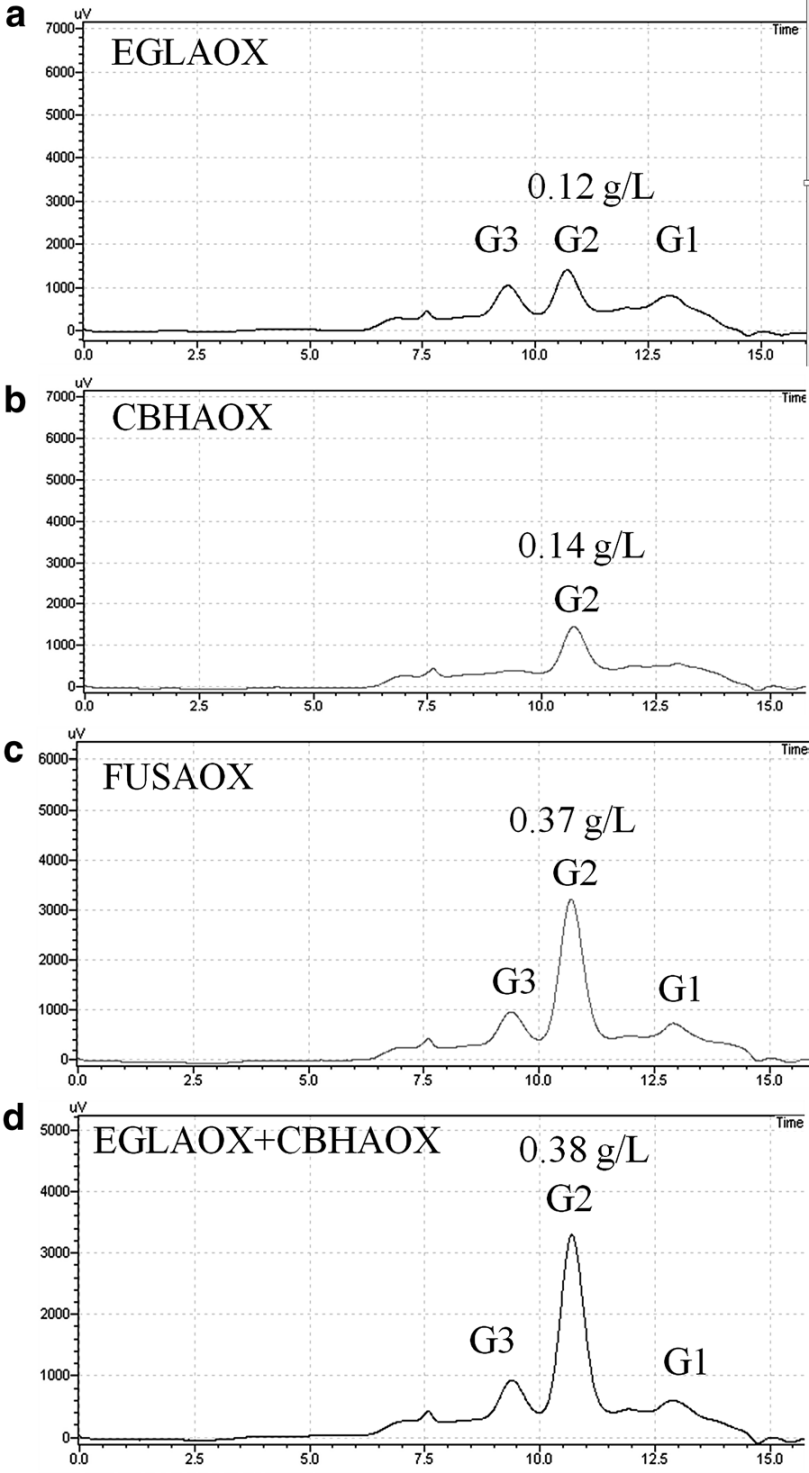
HPLC profiles of end products released by enzymatic hydrolysis of paper filter after incubation for 16 h at 50 °C. The supernatants from clones EGLAOX (**a**), CBHAOX (**b**), FUSAOX (**c**) or a combination of EGLAOX + CBHAOX (**d**) were incubated with filter paper and incubated for 16 h at 50 °C. Hydrolysis products were analyzed by HPLC. The concentration of cellobiose after the reaction is shown in each *graph*. *G1* glucose, *G2* cellobiose, *G3* cellotriose

We then evaluated specific cellulase activity of secreted EGII and CBHII by analyzing end product formation by HPLC after incubation with filter paper. As expected for an endocellulase, the main products from the reaction with EGLAOX were a mix of glucose, cellobiose and cellotriose (Fig. 7a) while CBHAOX (an exonuclease) only yielded cellobiose (Fig. 7b). The product profile from FUSAOX was a combination of the individual profiles from EGLAOX and CBHAOX but with a ~ threefold increase in the production of cellobiose (Fig. 7c) when compared with the reactions containing individual enzymes. A similar behavior was observed in an enzymatic assay in which equimolar amounts of EGLAOX and CBHAOX were combined (Fig. 7d).

## Discussion

In this work we sought to produce in *P. pastoris* an endoglucanase (EGII) and a cellobiohydrolase (CBHII) from *T. reesei* which represent highly expressed cellulases in this fungus (Goyal et al. 1991, Suominen et al. 1993; Vinzant et al. 2001). Although the individual production of EGII and CBHII in *P. pastoris* has been previously described (Boonvitthya et al. 2013) we investigated the coexpression of these enzymes as a protein fusion separated by the self-processing 2A peptide from FMDV in order to optimize cellulase production in this yeast. There are several reports of cellulase coexpression in yeasts with different outcomes. When Jeon et al. (2009) coexpressed a β-glucosidase 1 from *Saccharomycopsis fibuligera* and an endoglucanase from *Clostridium thermocellum* in *S. cerevisiae* a decrease of 23.8 and 33.11 % for BGase and CMCase activities were observed, respectively, when compared to the enzymes produced separately. Similarly, Den Hann et al. (2006) also observed a decrease of enzyme activity for coexpressed β-glucosidase 1 from *S. fibuligera* (37.67 %) and endoglucanase I from *T. reesei* (36.17 %). However, sometimes it was observed that coexpression has little (Wen et al. 2009) or no effect (Katahira et al. 2006) on cellulase activity. There are even reports of increased expression of enzymes when they are coexpressed (Fujita et al. (2004).

In our protein-fusion construct each cellulase bears its own native signal peptide since 2A cleavage occurs translationally. The position in which the genes were placed was intentionally determined—*cbhII* precedes *eglII* in order to facilitate detection in the CMC plate assay since the hydrolysis halo produced by endoglucanases is more evident than that produced by cellobiohydrolases. Thus, a simple verification of hydrolysis halo formation is an evidence that both enzymes are being produced (clone FUSAOX). The presence of a small hydrolysis halo around the negative control (pPIC9) has been routinely observed in other labs (personal communication) and might be due to a still uncharacterized cellulase-like activity since no cellulase genes have been annotated in the *P. pastoris* genome.

Two prominent protein species representing the two cellulases were produced by clone FUSAOX in a 1:1 ratio which is consistent with their co-translational production. For an efficient hydrolysis of lignocellulosic materials a different ratio might be desirable and this may be achieved just by adding further copies of either gene in the fusion construct in order to obtain an optimized enzyme blend. Furthermore, the higher molecular mass observed was confirmed to be the result of protein glycosylation which had been previously reported for these two enzymes (Boonvitthya et al. 2013). In general, the glycosylation of cellulases is related to the spatial separation between the catalytic domain and the cellulose binding domain (CBD), and protection of the linker from proteolytic cleavage (Srisodsuk et al. 1993; Kleywegt et al. 1997; Hui et al. 2002). It is reported that the yeast *P. pastoris* recognizes the same sequences for protein glycosylation as other eukaryotes but the pattern of sugar addition is different (Cabral et al. 2001).

The cellulase binding efficiency for the substrate is considerably increased by the presence of CBD and this increase is clearly correlated with a higher activity (Limon et al. 2001). As the 2A peptide is expected to be present at the C-terminal of the upstream protein partner after processing we investigated if the presence of the 33 amino acid residues extension might have affected the activity of CBHII, since the CBD is also located at the C-terminal portion of the enzyme. So far, there are only few reports in which the presence of the 2A linker at the C-terminal extension led to a small reduction in the enzymatic activity of the upstream protein (Ma and Mitra 2002; Ansari et al. 2004). Our results showed that CBHII activity was apparently not affect by the 2A peptide and the enzymes co-produced by clone FUSAOX acted synergistically on CMC yieldind mainly cellobiose. Riedel and Bronnenmeier (1998) produced in *E. coli* an artificial multienzyme consisting of a protein fusion of an exoglucanase and an endoglucanase from *Clostridium stercorarium*. The uncleaved fusion protein also showed a synergistic effect on Avicel releasing cellobiose and minor amounts of glucose and cellotriose. Likewise, Lee et al. (2012) produced in tobacco chloroplasts an endoglucanase from *Thermotoga maritima* and an exoglucanase from *Thermomonospora fusca* as a fusion protein separated by a 2A linker and observed similar results being cellobiose the main product when CMC was used as substrate.

In conclusion, the new system for cellulase production based on the 2A self-processing peptide prompts *P. pastoris* as an alternative platform for the production of heterologous cellulases for second-generation bioethanol.
